# Full-endoscopic resection of a thoracic facet joint osteoblastoma: A case report

**DOI:** 10.1016/j.bas.2026.106151

**Published:** 2026-06-25

**Authors:** Mohammad Badra, Hafez Saade, Fouad Assaf, Ramzi Moucharafieh, Youssef Jamaleddine

**Affiliations:** a Orthopedic Surgery and Traumatology, University of Balamand, Tripoli, Lebanon; b Clemenceau Medical Center, Beirut, Lebanon; c Department of orthopedic Surgery, Lebanese American University Medical Center-Rizk Hospital, Beirut, Lebanon

**Keywords:** Osteoblastoma, Thoracic spine, Facet joint tumor, Full-endoscopic spine surgery, Minimally invasive tumor resection, Case report

## Abstract

**Introduction:**

Osteoblastoma is a benign osteoid-forming tumor that often arises from the posterior spinal elements. Thoracic facet-joint involvement is uncommon, and open resection may require extensive soft-tissue dissection and threaten segmental stability. Full-endoscopic resection may reduce morbidity; however, its use for thoracic facet osteoblastoma has not previously been reported.

**Research question:**

Can full-endoscopic surgery achieve gross-total resection of a small, facet-centered thoracic osteoblastoma while preserving neurologic function and spinal stability?

**Materials and methods:**

We report a 26-year-old woman with 2 years of upper thoracic pain radiating to the anterior chest wall. MRI showed a right T4-T5 facet lesion with reactive paravertebral edema and no epidural extension or cord compression. CT confirmed a well-circumscribed 10 × 11 mm lytic lesion centered at the right T4-T5 facet joint and extending toward the T5 transverse process. Full-endoscopic posterior resection was performed through a 10-mm incision using endoscopic burrs and punches, with stepwise drilling to a circumferential bleeding bony boundary.

**Results:**

Histopathology confirmed osteoblastoma. The patient had immediate pain relief, mobilized on the day of surgery, and was discharged the next day without neurologic deficit. One-month CT demonstrated complete resection with no residual lesion. At 3 months, she remained pain-free with no new complaints.

**Discussion and conclusion:**

Full-endoscopic excision enabled gross-total resection and rapid recovery in this facet-centered thoracic osteoblastoma. Careful selection, precise localization, and controlled resection may achieve complete local tumor removal while limiting soft-tissue disruption and avoiding early radiographic or clinical evidence of instability.

## Highlights

• Thoracic facet-joint osteoblastoma was resected using a full-endoscopic posterior approach.
• Gross-total resection was achieved through a 10-mm incision.
• Neurologic function was preserved, with no early instability requiring fusion.
• The patient had immediate pain relief, same-day mobilization, and next-day discharge.
• This case supports endoscopic management in selected small posterior-element thoracic tumors.

## 1 Introduction

Osteoblastoma is a rare, benign osteoid-forming bone tumor, accounting for approximately 1% of primary bone tumors, that preferentially affects adolescents and young adults and frequently arises from the posterior elements of the spine (Galgano et al., 2016), (Limaiem et al., 2025). Clinical presentation is often nonspecific, with persistent axial pain and variable neurologic symptoms, which can delay diagnosis (Galgano et al., 2016), (Kan and Schmidt, 2008). CT best delineates osseous involvement, whereas MRI defines marrow and soft-tissue reaction and assesses epidural extension (Kan and Schmidt, 2008), (Wu et al., 2019). Because incomplete excision is associated with recurrence, gross-total resection remains the cornerstone of treatment when feasible (Galgano et al., 2016), (Jackson, 1978). In the spine, traditional open surgery may entail substantial soft-tissue dissection and bony exposure, with a considerable recovery burden (Ali et al., 2023). When stabilizing structures are compromised, fusion is often required (Elder et al., 2016). Full-endoscopic spine surgery provides a minimally invasive approach and has been increasingly applied in spinal oncology, but experience with benign primary thoracic tumors remains limited (Ali et al., 2023), (Sofoluke et al., 2022). A report suggests that fully endoscopic lumbar osteoblastoma resection can achieve gross-total excision while preserving stability (Newman et al., 2020). To the best of our knowledge, no case report or technical note has specifically described full-endoscopic resection of a thoracic osteoblastoma. We therefore present a thoracic T4-T5 facet-joint osteoblastoma treated using a full-endoscopic approach, highlighting operative strategy and early outcome.

## 2 Case presentation

A 26-year-old woman with no significant past medical history, presented to the clinic with a 2-year history of progressive upper thoracic back pain radiating to the anterior chest wall. She characterized the pain as deep, aching, and moderate in intensity, with prominent nocturnal exacerbation. She denied antecedent trauma, fever, night sweats, or unintentional weight loss. Initial treatment was conservative (nonsteroidal anti-inflammatory drugs, analgesics, and physiotherapy) for presumed muscular etiology, with partial symptoms improvement while taking NSAIDs but consistent recurrence upon discontinuation.

**Fig. 1:**
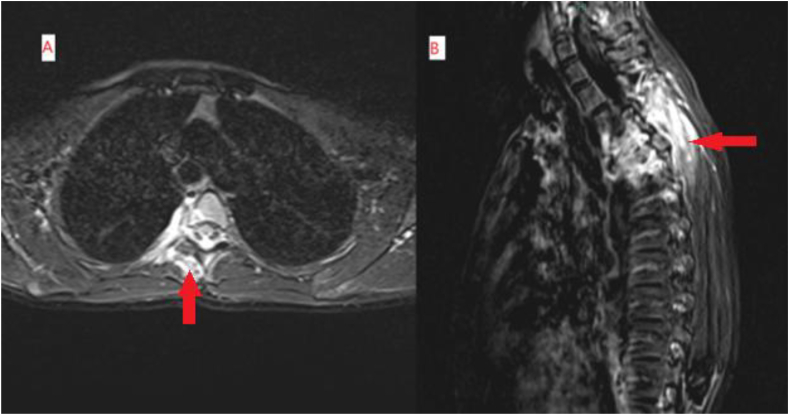
Magnetic resonance imaging (MRI) of the thoracic spine demonstrating a focal lesion centered at the right T4-5 facet joint, with surrounding paravertebral muscle edema (red arrows), without epidural extension or spinal cord compression. (A: axial cut; B: sagittal cut).

**Fig. 2:**
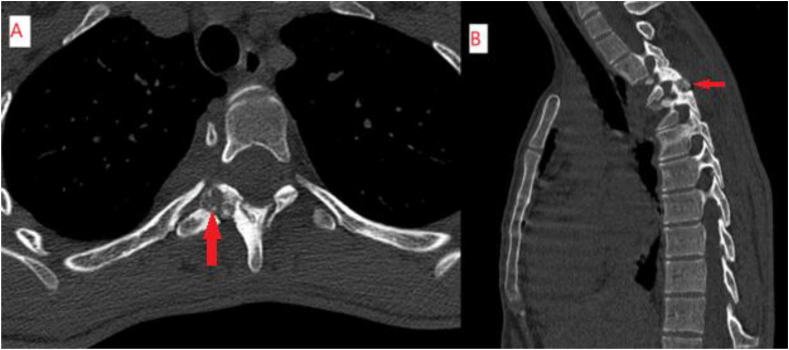
Computed tomography (CT) of the thoracic spine showing a well-defined lytic lesion (10 × 11 mm; red arrows) predominantly involving the right T4-5 facet joint, with extension toward the origin of the right T5 transverse process, consistent with an osteoblastoma pattern. (A: axial cut; B: sagittal cut).

Magnetic resonance imaging (MRI) of the thoracic spine demonstrated a focal lesion arising from the right T4-5 facet joint, with high signal intensity involving the right posterior elements and associated paravertebral muscle edema (Fig. 1). There was no epidural extension, spinal canal compromise, or spinal cord compression. Computed tomography (CT) further characterized a well-defined lytic lesion measuring 10 × 11 mm centered in the right T4-5 facet joint, extending into the origin of the right T5 transverse process (Fig. 2). Overall, the imaging appearance was highly suggestive of osteoblastoma with reactive inflammatory changes.

After discussion with the patient, surgical management was recommended given persistent symptoms and localized pathology. Considering the patient's young age, the absence of radiologic instability, and the lesion's small unilateral facet-centered location, a full-endoscopic posterior resection was planned as a fusion-sparing approach to minimize soft-tissue disruption.

**Fig. 3:**
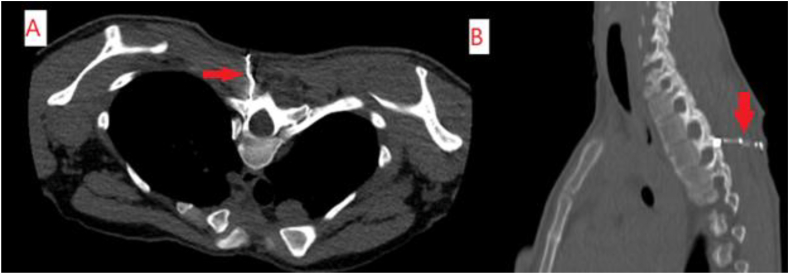
Preoperative CT-guided placement of a metallic localization wire (red arrows) into the left T4 pedicle to facilitate accurate intraoperative level identification. (A: axial cut; B: sagittal cut).

**Fig. 4:**
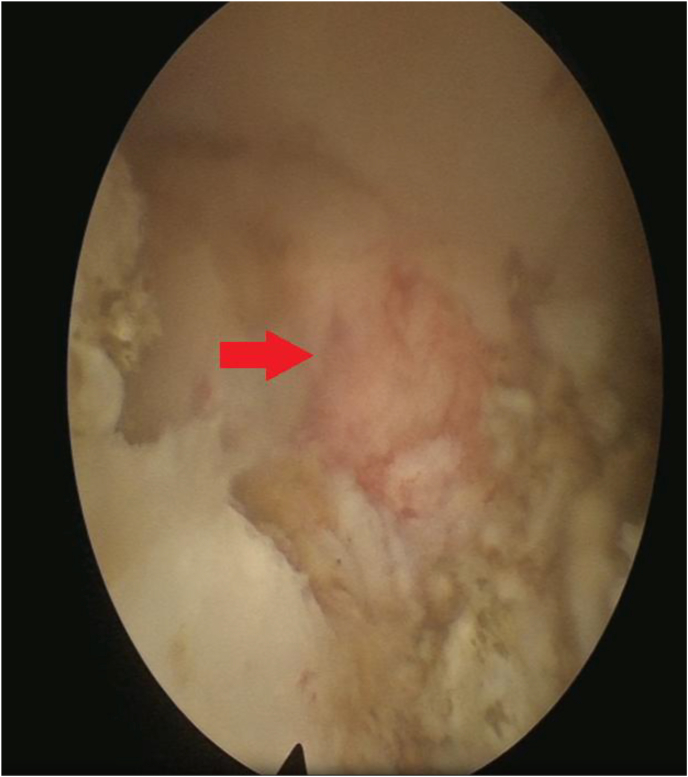
Intraoperative endoscopic view showing tumor at the right T4-5 facet region.

**Fig. 5:**
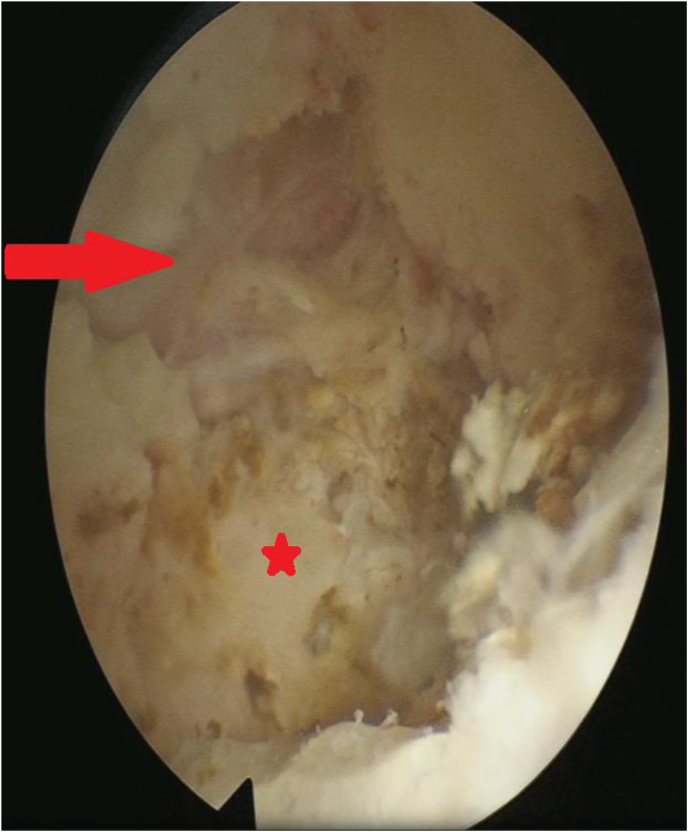
Endoscopic view after lesion resection demonstrating the lateral dural margin (red arrow) and the dorsal aspect of the T5 rib (red asterisk), confirming the depth and extent of bony resection.

**Fig. 6:**
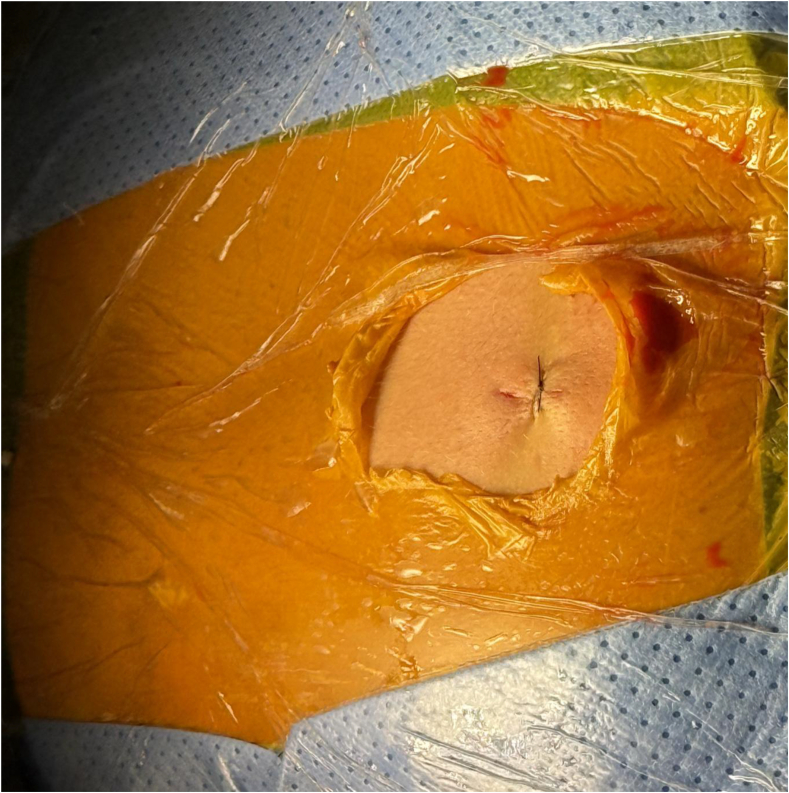
Final skin closure following full-endoscopic posterior resection, demonstrating a minimal incision closed with a single suture, without drain placement.

For accurate intraoperative level identification, a metallic localization wire was placed preoperatively into the left T4 pedicle under CT guidance (Fig. 3). Under general anesthesia, the patient was positioned prone on a radiolucent table. Using fluoroscopic guidance, the right T4-5 level was confirmed. Using the RIWOspine endoscopic system, a 10-mm skin incision was made lateral to the midline over the right T4-5 facet joint. The dilator was docked onto the facet joint, followed by insertion of the working cannula. Under endoscopic visualization, the inferior aspect of the right T4 lamina, the superior aspect of the right T5 lamina, and the right T4-5 facet joint were exposed. Tumor was readily identified and removed using endoscopic burrs, punches, and radiofrequency ablation (Fig. 4). Resection proceeded in a piecemeal fashion until circumferential bleeding bone margins were achieved. The inferior articular process of T4, the superior articular process of T5, the diseased medial portion of the T5 transverse process, and part of the right T4 lamina were resected. The lateral dural margin and the dorsal aspect of the T5 rib were exposed (Fig. 5). Hemostasis was secured using standard bipolar electrocautery under endoscopic visualization, and the wound was closed with a single suture without placement of a drain (Fig. 6). The total operative time was 119 min.

**Fig. 7:**
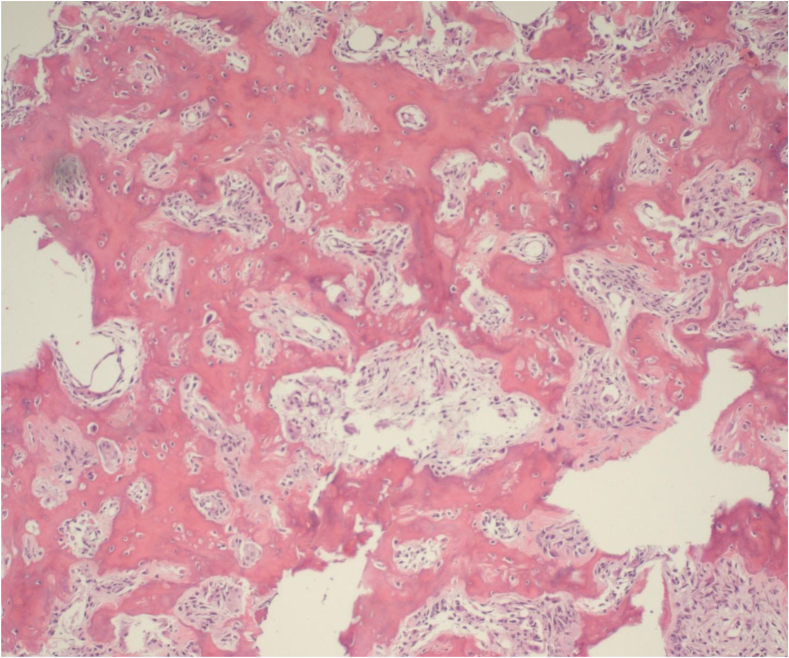
Histopathological examination of the resected specimen demonstrating interlacing trabeculae of woven bone and osteoid lined by prominent osteoblasts within a highly vascular fibrovascular stroma, consistent with osteoblastoma.

Histopathological examination showed interlacing trabeculae of woven bone and osteoid lined by prominent, plump osteoblasts within a highly vascular loose fibrovascular stroma, without significant cytologic atypia or increased mitotic activity (Fig. 7), confirming osteoblastoma.

**Fig. 8:**
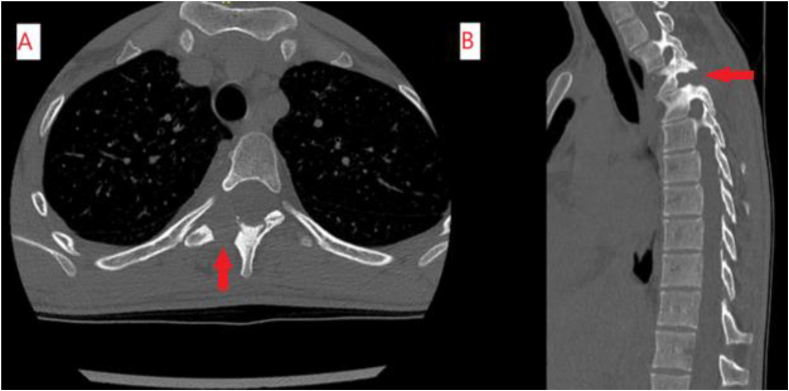
Follow-up CT scan at 1 month postoperatively demonstrating complete resection of the lesion with no residual tumor (red arrows) at the right T4-5 facet joint region. (A: axial cut; B: sagittal cut).

Postoperatively, the patient reported immediate relief of her upper thoracic pain. She was mobilized on the day of surgery and discharged home the following day with oral analgesia (paracetamol as needed). A postoperative CT scan obtained 1 month after surgery demonstrated complete resection of the lesion (Fig. 8). At 3 months of clinical follow-up, the patient remained pain-free and reported no new complaints.

## 3 Discussion

Osteoblastoma is an uncommon benign osteoid-forming tumor with a predilection for the posterior elements of the spine (Boriani et al., 1992). Although benign by histology, spinal lesions can be locally aggressive and are a well-described cause of prolonged, nonspecific axial pain that may be treated symptomatically for months to years before definitive imaging establishes the diagnosis (Boriani et al., 1992). In classic spine series, posterior elements and pedicles were more frequently involved than the vertebral body, making facet- and pars-based lesions common surgical targets (Boriani et al., 1992). The present case, an osteoblastoma centered in the T4-5 facet complex, illustrates how a small posterior-element lesion can drive substantial symptoms and inflammatory changes while remaining neurologically silent.

Multimodal imaging is central to diagnosis and operative planning. CT best defines the bony epicenter, cortical breach, and the true osseous margins of posterior-element lesions, whereas MRI characterizes marrow and paraspinal soft-tissue reaction and critically assesses epidural extension or spinal cord compromise (Shaikh et al., 1999), (Liu et al., 2020). Prior imaging studies emphasize that bone marrow edema and soft-tissue edema on MRI may be extensive, such that CT is often required to confirm and precisely localize the osseous focus (Liu et al., 2020). In our patient, MRI demonstrated prominent paravertebral muscle edema without epidural involvement, while CT localized a well-circumscribed 10 × 11 mm lytic lesion at the right T4-5 facet region, enabling a targeted surgical approach.

An important diagnostic consideration is the overlap between osteoid osteoma and osteoblastoma. Size-based concepts are commonly used with osteoid osteoma classically ≤1 cm and osteoblastoma ≥2 cm, with an intermediate “gray zone”, but histology and behavior remain decisive and small osteoblastomas can occur (Yalcinkaya et al., 2014). Our lesion lies near this gray zone, reinforcing that size alone should not exclude osteoblastoma when imaging and symptoms suggest a benign osteoid-producing process and when a tissue diagnosis is required to guide definitive therapy. In this case, the final diagnosis favored osteoblastoma on the basis of histopathologic findings in conjunction with the clinical and imaging features, rather than lesion size alone.

Surgical excision remains the mainstay for symptomatic spinal osteoblastoma because incomplete removal is associated with local recurrence. In a study (Boriani et al., 2012) that explicitly stratified outcomes by whether the lesion was “intact”, meaning the index (first) definitive treatment was performed by their team, versus “non-intact”, referring to tumors previously subjected to open biopsy or prior unsuccessful surgery elsewhere, often presenting as recurrent or progressive disease. Recurrence was significantly more frequent in the non-intact cohort than in the intact cohort, underscoring the prognostic importance of the first operation and the need for careful margin strategy and long-term surveillance (Boriani et al., 2012). Their series also highlights a practical perioperative consideration.

For posterior-element tumors of the thoracic spine, the surgical aim is complete excision while preserving biomechanical stability. The thoracic spine is biomechanically distinct from the cervical and lumbar regions because stability is conferred not only by the posterior osseoligamentous structures but also by the rib cage and sternocostovertebral articulations (Oda et al., 1996), (Brasiliense et al., 2011), (Liebsch and Wilke, 2022). Experimental data further suggest that, with the rib cage intact, unilateral thoracic decompression including facetectomy does not necessarily produce significant immediate destabilization, particularly at the level of the true ribs (Healy et al., 2014). Consistent with this, a clinical series reported that unilateral total facetectomy without instrumented fusion for thoracic dumbbell tumors did not lead to postoperative deformity or instability requiring additional surgery at mid-to long-term follow-up (Ishikawa et al., 2023). In the present case, the lesion was small, unilateral, and centered in the facet and posterior elements, making a fusion-sparing resection reasonable. Nevertheless, because this is a single case with short follow-up, preservation of segmental stability should be interpreted cautiously and not as definitive proof of long-term biomechanical preservation. When resection necessarily compromises stabilizing structures, instrumentation may be required to restore stability (Li et al., 2013). In non-oncologic spine surgery studies, open posterior midline approaches provide broad exposure, but they typically involve substantial paraspinal muscle detachment and retraction, contributing to greater approach-related soft-tissue morbidity compared with muscle-sparing technique (Chang et al., 2018), (Street et al., 2016). In contrast, full-endoscopic posterior techniques leverage a small working corridor and magnified visualization to limit paraspinal muscle disruption, in lumbar decompression surgery, with objective MRI-based comparisons demonstrate significantly reduced approach trauma and less paraspinal muscle fatty change with endoscopic techniques relative to traditional open translaminar microsurgery (Hellinger et al., 2022). Thoracic endoscopy, however, is anatomically unforgiving, safe access depends on careful trajectory planning around level-specific foraminal or interlaminar constraints and adherence to established safety boundaries and anatomic limits (Choi and Munoz-Suarez, 2020). Endoscopic tumor surgery remains an evolving field. A systematic review of full-endoscopic spinal oncology reported that gross-total resection is frequently achievable in selected extradural tumors, with generally low blood loss and short hospital stays, while emphasizing that the current evidence is predominantly case reports and small case series (Tummala et al., 2025).

Our case adds practical support for a full-endoscopic posterior surgery in a small, facet-centered thoracic osteoblastoma. Several technical elements were central. First, accurate level localization was secured using a preoperative CT-guided marker to mitigate wrong-level risk in the upper thoracic spine. Second, resection was performed with controlled drilling through an endoscope, removing tumor in a piecemeal fashion until circumferential bleeding bone margins were obtained. Third, bone removal was intentionally limited to what was required tumor excision to preserve postoperative segmental stability. This experience aligns with full endoscopic osteoblastoma excision in the lumbar spine with intraoperative confirmation of total excision (Newman et al., 2020). While thoracic and lumbar spine differ, the shared concept is that posterior-element, facet-centered tumors are attractive candidates for endoscopic management when safe margins can be achieved without destabilizing the motion segment.

We would consider a full-endoscopic posterior approach most appropriate for carefully selected benign posterior-element spinal lesions that are small, unilateral, well circumscribed, and centered in the facet joint, lamina, pars, or adjacent posterior elements, particularly when there is no radiologic instability, deformity, substantial epidural extension, cord compression, or close involvement of critical neural or vascular structures. The lesion should also appear amenable to complete local excision through a limited working corridor without requiring destabilizing bone removal. In the thoracic spine, the relative inherent stability provided by the rib cage and costovertebral articulations may further support a fusion-sparing strategy in selected cases, although this should not replace individualized assessment of preoperative stability and the extent of planned bone removal. Faced with a comparable lesion in the future, namely a small unilateral thoracic facet-centered benign tumor without epidural disease or instability, we would again consider a full-endoscopic, fusion-sparing approach, provided that accurate level localization is possible and that conversion to an open or instrumented procedure remains available if adequate resection or stability cannot be safely maintained.

The principal limitation of this report is the short follow-up, with radiographic follow-up available at 1 month and clinical follow-up at 3 months. Longer follow-up is needed to confirm the preservation of segmental stability. Osteoblastoma recurrence can occur late, and long-term surveillance is warranted even after apparent complete excision (Galgano et al., 2016). In addition, endoscopic tumor surgery should remain reserved for carefully selected lesions; tumors with substantial epidural soft-tissue extension, proximity to critical neural or vascular structures, or a need for wide en bloc resection with negative margins may be better treated with open or combined approaches.

## 4 Conclusion

Full-endoscopic posterior excision of a facet-centered thoracic osteoblastoma achieved gross-total resection, rapid pain resolution, and next-day discharge without neurologic deficit. Precise level localization and stepwise drilling to circumferential bleeding bone margins enabled tumor excision while limiting paraspinal disruption with the aim of preserving postoperative segmental stability. This case supports full-endoscopic management as a feasible option for small, posterior-element thoracic osteoblastomas in carefully selected patients. Longer surveillance and larger series are needed to define recurrence rates and refine indications.

## Author contributions

Conceptualization: YJ, MB. Image extraction: MB, HS, FA. Writing original draft: YJ. Writing-review and editing: all authors. Final approval of the manuscript: all authors.

## Funding

This research did not receive any specific grant from funding agencies in the public, commercial, or not-for-profit sectors.

## Declaration of competing interest

The authors declare that they have no known competing financial interests or personal relationships that could have appeared to influence the work reported in this paper.
